# Genome-Wide Scanning for Signatures of Selection Revealed the Putative Genomic Regions and Candidate Genes Controlling Milk Composition and Coat Color Traits in Sahiwal Cattle

**DOI:** 10.3389/fgene.2021.699422

**Published:** 2021-07-09

**Authors:** Satish Kumar Illa, Sabyasachi Mukherjee, Sapna Nath, Anupama Mukherjee

**Affiliations:** ^1^Division of Animal Genetics and Breeding, Indian Council of Agricultural Research-National Dairy Research Institute, Karnal, India; ^2^Artificial Breeding Research Center, Indian Council of Agricultural Research-National Dairy Research Institute, Karnal, India

**Keywords:** indigenous cattle, SNP genotyping, selection signatures, gene identification, DCMS, gene

## Abstract

**Background:**

In the evolutionary time scale, selection shapes the genetic variation and alters the architecture of genome in the organisms. Selection leaves detectable signatures at the genomic coordinates that provide clues about the protein-coding regions. Sahiwal is a valuable indicine cattle adapted to tropical environments with desirable milk attributes. Insights into the genomic regions under putative selection may reveal the molecular mechanisms affecting the quantitative and other important traits. To understand this, the present investigation was undertaken to explore signatures of selection in the genome of Sahiwal cattle using a medium-density genotyping INDUS chip.

**Result:**

De-correlated composite of multiple selection signals (DCMS), which combines five different univariate statistics, was computed in the dataset to detect the signatures of selection in the Sahiwal genome. Gene annotations, Quantitative Trait Loci (QTL) enrichment, and functional analyses were carried out for the identification of significant genomic regions. A total of 117 genes were identified, which affect a number of important economic traits. The QTL enrichment analysis highlighted 14 significant [False Discovery Rate (FDR)-corrected *p*-value ≤ 0.05] regions on chromosomes BTA 1, 3, 6, 11, 20, and 21. The top three enriched QTLs were found on BTA 6, 20, and 23, which are associated with exterior, health, milk production, and reproduction traits. The present study on selection signatures revealed some key genes related with coat color (*PDGFRA, KIT*, and *KDR*), facial pigmentation (*LEF*), milk fat percent (*MAP3K1, HADH, CYP2U1*, and *SGMS2*), sperm membrane integrity (*OSTC*), lactation persistency (*MRPS30, NNT, CCL28, HMGCS1, NIM1K, ZNF131*, and *CCDC152*), milk yield (*GHR* and *ZNF469*), reproduction (*NKX2-1* and *DENND1A*), and bovine tuberculosis susceptibility (*RNF144B* and *PAPSS1*). Further analysis of candidate gene prioritization identified four hub genes, viz., *KIT, KDR, MAP3K1*, and *LEF*, which play a role in coat color, facial pigmentation, and milk fat percentage in cattle. Gene enrichment analysis revealed significant Gene ontology (GO) terms related to breed-specific coat color and milk fat percent.

**Conclusion:**

The key candidate genes and putative genomic regions associated with economic traits were identified in Sahiwal using single nucleotide polymorphism data and the DCMS method. It revealed selection for milk production, coat color, and adaptability to tropical climate. The knowledge about signatures of selection and candidate genes affecting phenotypes have provided a background information that can be further utilized to understand the underlying mechanism involved in these traits in Sahiwal cattle.

## Introduction

Sahiwal is a well-known breed belonging to the humped zebu cattle group (*Bos indicus*), having its origin in the northwestern region of the Indian subcontinent (Muhuyi et al., 1999). Apart from their high milk-producing ability, these cattle breed also possesses unique adaptability to hot and humid climate prevailing in their native tract and their known resistance to tropical disease, ticks, and parasites (Singh et al., 2005). The milk of this cattle yields high fat (4.6–5.2%) and solid non-fat (SNF: 8.9–9.3%) (Joshi et al., 2001). Sahiwal cattle are utilized in crossbreeding programs around the world because of their higher milk production qualities and endurance to harsh environments (Maule, 1990). Such crossbreeding work has affected the numbers and distribution of indigenous purebred cattle to some extent in recent years (DAHDF,2018/2019). In view of this, the Government of India established purebred cattle nucleus herds under All India Coordinated Research Project (AICRP). The major objectives of this AICRP are to conserve the valuable Sahiwal germplasm and to continue the genetic improvement program mostly for production traits.

It is now accepted broadly that the modern Zebu cattle were domesticated in the Indus valley approximately 6,800 years ago from Aurochs (*Bos primigenius nomadicus*) and was later introduced to different parts of tropical regions (Ajmone-Marsan et al., 2010). Domestication of livestock benefitted humankind in terms of milk, meat, and draft power. Selective breeding and genetic isolation help in formation of numerous cattle breeds and facilitates in maintaining the diversity of genome resources and retain the characteristics of adaptation to local environments (Felius, 1995). Artificial selection increases the beneficial alleles related to economic traits and aids in improving the production parameters (Diamond, 2002; Flori et al., 2009). The process of domestication and breed formation in mammals levied a constant source of selection pressure on a divergent variety of traits in all the domesticated species and left detectable impressions at individual genomes. These genomic regions provide straightforward clues about the functional variants concerning the traits (Andersson and Georges, 2004; Oleksyk et al., 2010). The advent of the cost-effective genotyping allowed more individuals to be genotyped with dense single nucleotide polymorphism (SNP) array and thereby facilitating the precise identification of the genomic regions of livestock with better resolution and accuracy (Jensen et al., 2016). These developments also helped in the mapping of signatures under selection at the genome level in *Homo sapiens* and other species of animals (Mathieson et al., 2015; Moon et al., 2015). Selection signatures are specific variations at the DNA level that arise due to changes in the genomes of both selected and neutral loci of a species that has undergone selection over the years (Kreitman, 2000). Several statistical models were developed to determine the signatures of selection in recent years. Variants under selection pressure generate the typical genomic patterns such as (i) change in the allele frequency spectrum (either low or high frequencies); (ii) greater number of homozygous genotypes; (iii) long haplotypes are most common; and/or (iv) intense differentiation of local population. Several studies were conducted utilizing more than one statistic, viz., Integrated Haplotype Score (iHS), Cross-Population Extended Haplotype Homozygosity (XP-EHH), and Fixation index (F_*ST*_) to detect the signatures of selection that exploit the advantage of complementarity of methods, intending to improve the statistical power (Weir and Cockerham, 1984; Voight et al., 2006). A new method was proposed in which different *p-*values are combined to give a composite of signals (CMS) for the first time (Grossman et al., 2010). Few studies suggested the use of combining different selection signals such as meta-SS and Composite Selection Signals (Utsunomiya et al., 2013; Randhawa et al., 2016). However, many of these earlier studies did not consider the covariance among the statistics, and consequently, a new method of composite signals was proposed where the outputs of different methods were combined and accounted for the covariance between the statistics. It differed from the other composite statistics by considering the dependencies among the univariate statistics and was termed as De-correlated composite of multiple selection signals (DCMS) (Ma et al., 2015; Lotterhos et al., 2017). This statistic is found to be more effective than earlier methods and helps in prioritizing the candidate genes affecting major economic traits in various species that are helpful to medicine, agriculture, and animal breeding. It is preferred over the univariate statistics as the latter retains high local resolution. Besides these beneficial attributes, DCMS estimation is also possible with better accuracy even with less demography information. Selection signature analysis in Russian and Swedish local cattle using de-correlated DCMS revealed functional variants under selection related to production, reproduction, and adaptation (Yurchenko et al., 2018; Ghoreishifar et al., 2020).

Under the vast physio-geographical region of the Indian subcontinent, Sahiwal is a valuable *indicine* cattle adapted to tropical environments with desirable milk attributes, and it is apparent that the genomic regions of Sahiwal cattle will be under intense putative selection for centuries. Literature on these aspects is still scanty so far. Therefore, to understand the molecular mechanism affecting the quantitative and other important traits in these cattle, the present investigation was undertaken to explore signatures of selection in the genome of Sahiwal cattle. Simultaneously, annotation of genes and quantitative trait loci was also carried out for prioritizing the candidate genes having major effects on various production and adaptive traits including coat color in Sahiwal cattle using the medium-density genotyping assay with INDUSCHIP2, curated from Illumina BovineSNP50 Bead Chip and developed by the National Dairy Development Board (NDDB) for genotyping indicine cattle (NDDB, 2019).

## Materials and Methods

### Animals and Genotyping

The study was conducted on 193 Sahiwal cattle belonging to the germplasm unit of AICRP maintained at ICAR-National Dairy Research Institute, Karnal, India. The entire population of Sahiwal cattle was further categorized into two subpopulations, viz., founder/unrelated animals (*n* = 41) and those farm-born/related (*n* = 152). These farm-born animals were born in the germplasm unit between 2003 and 2016. All the cattle were genotyped with INDUSCHIP2 consisting of 53,648 SNPs. This genotyping chipset is developed by the NDDB, India, which is customized from commercially available Illumina Bovine SNP chip (BovineSNP50K v3 Bead Chip) to genotype native cattle breeds and their crosses for implementing the genomic selection schemes in small and organized herds in India (NDDB, 2019).

The quality control (QC) of SNP data was implemented in Plink v1.9 program (Purcell et al., 2007). SNPs with a genotype call rate lower than 0.95 and a minor allele frequency less than 0.05 and those SNPs with Hardy–Weinberg equilibrium below 0.001 were removed. In addition, SNPs with duplicated position, located on the sex chromosome and with an unidentified position on UMD3.1 assembly, were excluded using the –exclude option. Furthermore, the highly related individual information was obtained from the –genome command in the form of PI-HAT indices. Individual pairs with unusually high PI-HAT values were discarded from the analysis to minimize the bias of sample size. Quality control of genotypes was again performed for phasing of haplotypes with the *Shapeit* program (Delaneau et al., 2012) to get high-quality SNPs. In total, 37,594 SNPs were considered after QC for final analysis.

### Principal Component Analysis

Principal component analysis (PCA) was carried out in R environment with the *snprelate* package (Zheng et al., 2012) to explore the structure and clustering of the samples with the help of plotting the genotypes of all the individuals, belonging to two subpopulations of Sahiwal cattle.

### De-Correlated Composite of Multiple Selection Signals

As outlined by Yurchenko et al. (2018), the current study used the DCMS method to combine all the five statistics, viz., F_*ST*_, Haplotype Homozygosity (H1), Modified Haplotype Homozygosity (H12), Tajima’s D index, and Nucleotide diversity (pi) (Nei and Li, 1979; Weir and Cockerham, 1984; Tajima, 1989; Garud et al., 2015). The DCMS statistic is computed at any given loci *l* as follows:

$$D\,C\,M\,S_{l}=\sum_{t=1}^{n}\frac{l\,o\,g\,\left[\frac{1-p_{l\,t}}{p_{l\,t}}\right]}{\sum_{i=1}^{n}\left|r_{i\,t}\right|}$$

Here, *p*_*lt*_ refers to the *p-*value at the *l* position for each statistic *t*. The denominator consists of a correlation component (*r*_*it*_), which is the weighing factor at each locus. The weighing factors were genome-wide correlations between all the univariate statistic pairs (Ma et al., 2015); however, the statistic with greater correlation adds less to the calculation. To obtain the DCMS, all the statistics were converted into *p-*values using one-tailed and two-tailed ranks, where these fractional ranks lie between 1/(*n* + 1) and *n*/(*n* + 1), respectively.

#### Fixation Index

Fixation index is known as a measure of population differentiation, and it was calculated for each SNP.

For the purpose of estimation of F_*ST*_ in our study, we have divided the entire Sahiwal population (*n* = 193) into two subpopulations, viz., founder/unrelated animals (*n* = 41) and farm-born/related animals (*n* = 152) based on the estimated average amount of IBD values sharing across all loci, i.e., pairwise relatedness. This is accomplished because F_*ST*_ is a parameter that measures genetic structure in a subdivided population. As per Wright (1951), F_*ST*_ is also the probability that alleles drawn randomly from a subpopulation are “identical by decent” (IBD), relative to an ancestral population.

The F_*ST*_ analysis was carried out between these two subpopulations of Sahiwal cattle using the–fst and–within functions of Plink1.9. Zero F_*ST*_ values were converted to zeros and the F_*ST*_ values of each SNP were smoothed with the *runmed* function in the R program.

After estimation of F_*ST*_ values, the other univariate statistics such as haplotype homozygosity (H1), modified haplotype homozygosity (H12), Tajima’s D index, and Nucleotide Diversity (pi) were estimated. Subsequently, all the five statistics were combined into one single framework of DCMS.

#### Haplotype Homozygosity Statistics (H1 and H12)

Phasing of each chromosome was carried out separately with the SHAPEIT2 *version* program (Delaneau et al., 2012) with default parameters like conditional states (–states 400) and the effective population (–effective-size 108), which was calculated using SNePV1.1 program with the default parameters (Barbato et al., 2015). A bovine recombination map was used to rectify the variation due to the recombination rate along the autosomal genome (Ma et al., 2015). An R script was used to convert the phased haplotypes into the format as required by the H12_H1H2.py program^1^. H1 and H12 statistics were obtained for all the SNPs by using the parameters of window size of 14 SNPs and step size of 1 (-window 14 -jump 1) (Garud et al., 2015).

#### Tajima’s D and Nucleotide Diversity (π)

The vcftools program was used to compute Tajima’s D and π (*pi*) statistics (Danecek et al., 2011); both statistics were calculated for each chromosome separately (Danecek et al., 2011). Tajima’s D index was obtained with the parameter of non-overlapping sliding windows of 300 MB (–Tajima D 300). The *p-*values were assigned to each SNP within the bin. All the missing values were changed to zero. The *pi* values for all SNPs were computed with the function –site-pi function. The raw *p-*values were smoothed with the *runmed* function implemented in the R program with a window of 31 SNPs and constant end rule (*k* = 31; endrule = “constant”) as described by Yurchenko et al. (2018).

#### DCMS Estimation

All five statistic parameters (H1, H12, Tajima’s D index, π, and F_*ST*_) for each SNP were combined to a new composite signal as DCMS. Based on the functional ranks, the left-tailed test was applied to Tajima’s D values and π, and the right-tailed test was applied to H1 and H12 and F_*ST*_ statistic, respectively, using stat_to_p-value function in the MINOTAUR package in R environment (Verity et al., 2017). Later, the covNAMcd function (alpha = 0.75, nsamp = 50,000) was applied from the rrcovNA R package (Todorov et al., 2011) and a correlation matrix of *n* × *n* order was calculated, which will be input to DCMS function of MINOTAUR R package (Verity et al., 2017) and the genome-wide DCMS values were computed. These DCMS values were transformed to a normal distribution with the robust linear model (*rlm*) using the MASS R package (Venables and Ripley, 2002) as outlined in Yurchenko et al. (2018). Then, DCMS statistics fitted to normal distribution were transformed to *p*-values by the *pnorm* function (lower.tail = FALSE, log.p = FALSE). Finally, thus obtained *p*-values were converted into the respective *q*-values after the Benjamini and Hochberg (1995) correction using the *q-*value R function (Storey and Tibshirani, 2003). The false discovery rate is calculated, which minimizes the error rate from multiple tests.

### Identification of Functional Genes and QTL

The genomic regions were considered as significant if *q*-value is lower than 0.05 for adjacent SNPs. The boundaries of the genomic regions were determined from the SNP with a *q* value greater than 0.1. The gene and QTL annotations were performed using R package GALLO (Genomic Annotation in Livestock for positional candidate Loci) (Fonseca et al., 2020). The gene and QTL annotation files (.gtf and .gff files) derived from the ARS-UCD1.2 assembly (Rosen et al., 2018) and Animal QTL Database (Hu et al., 2013) were used for the gene and QTL identification, respectively. The QTL enrichment analysis was also performed for all the QTLs annotated by the chromosome-based method using the same GALLO package. A bootstrap method was implemented to correlate the observed and expected number of QTLs per trait from the cattle QTL database with 1,000 iterations of random sampling. The calculated *p-*values in the enrichment analysis were also adjusted using FDR (<5%) for multiple testing.

### Prioritization of Candidate Genes and Gene Enrichment Analysis

The candidate gene prioritization analysis was implemented in Topp Gene Suite (Chen et al., 2009), and the program requires a training set and a test set of genes. The training set of genes were obtained from GUILDify software by providing the keywords “sperm plasma membrane integrity,” “milk yield,” “milk fat percent,” “lactation persistency,” “facial pigmentation,” “eye area pigmentation,” “body weight,” “maternal behavior,” and “Bovine Tuberculosis Susceptibility.” The gene list obtained from this analysis was used as an input in Topp Gene Suite and the identified genes were considered as a test gene set. The prioritization analysis is a multivariate method that utilizes the functional information from the Gene Ontology terms, human and mouse phenotypes, PubMed publications, and diseases. The significant *p*-values were obtained by linking all the *p*-values of a random sample of 5,000 genes. The candidate genes were prioritized after adjustment of *p-*values with FDR ≤ 5%. The prioritized genes were considered as an input in *NetworkAnalystv.3.0* (Zhou et al., 2019). A protein–protein interaction network was also generated, which is based on the string protein–protein interaction database with a confidence score cutoff of 900. Networks with nodes and edges were generated, and the networks with gene ontology terms such as Molecular function, Biological process, and Cellular components were also produced.

## Results

### Quality Control and PCA

Quality control of genotypes for minor allele frequency, genotype call rate, Hardy–Weinberg equilibrium, and duplicated genotype parameters had excluded 12,842 SNPs and left the final dataset with 37,594 genotypes. The effective population size was calculated as 52 in Sahiwal population based on genotype data using SNeP V1.1 software.

The PCA was performed in the final dataset and showed that all the individuals in the dataset were homogeneous. Hence, the selection signature analysis was carried out by considering all the individuals as one group after exclusion of related individuals from the analysis with the help of the –genome command in Plink. This function had excluded 41 related individuals from the original dataset, having pairwise PI_HAT values above 0.1. Final analysis was performed on 152 individuals from the final dataset with 37,594 SNPs.

### De-Correlated Composite of Multiple Selection Signals

The DCMS values were calculated for all 37,594 SNPs; the *p-*values were corrected (FDR < 0.05) and fitted to normal distribution. The selection analysis revealed 14 significant genomic regions with their average length observed as 652.06 ± 830.18 KB, ranging from 60.05 to 3,444.44 KB length. The total size of the genomic region is 9.78 Mb, with 117 total number of protein-coding regions found in the study (Table 1). Our study identified the four most significant genomic regions on BTA 6 (17567590:18290048, *q-*value = 1.76E-08), BTA 20 (30375415:30375415, *q-*value = 8.73E-07; 22144338:22425353, *q-*value = 2.68E-05), and BTA 23 (39175206:39671814, *q-*value = 1.79E-08), respectively (Table 1). The findings of gene annotation (Table 2) revealed a wide range of genes related to different types of traits, viz., growth (*CPNE4* and *RALGAPA1*), survival (*MIER3*), adaptation (*GIPC2*), heat tolerance (*DNAJB4*), milk protein content (*MRPL3, NUDT16*, and *NEK11*), milk yield (*ADGRL4* and *PTGFR*), milk production (*RPS6KA2, ZNF469*, and *GHR*), milk fat secretion (*HADH, CYP2U1, SGMS2, SLC25A21*, and *MAP3K1*), carcass traits (*COL25A1*), sperm membrane integrity (*OSTC*), resistance to bovine tuberculosis and Johne’s disease (*RNF144B* and *PAPSS1*), coat color (*PDGFRA, KIT*, and *KDR*), eye area pigmentation (LEF1), reproduction (*DENND1A* and *NKX2-1*), lactation persistency (*MRPS30, NNT, CCL28, HMGCS1, NIM1K, ZNF131*, and *CCDC152*), body height (*NHLRC1*), and mineral concentration (*FAM8A1*).

**TABLE 1:** Summary of the genomic regions determined by the de-correlated composite of multiple selection signals (DCMS) in Sahiwal cattle.

Breed	*N* regions	Average ± SD (KB)	Min (KB)	Max (MB)	*N* SNP	Total size (Mb)	*N* genes
Sahiwal	14	652.06 ± 830.18	60.05	3.44	221	9.78	117

**N* Regions, number of segments identified using the DCMS method as significant genomic regions harboring signatures of selection; Min, minimum size of significant genomic regions; Max, maximum size of significant genomic regions; *N* SNPs, number of SNPs identified within the regions detected as signatures of selection using the DCMS method (*q*-value < 0.05); *N* genes, number of protein coding genes identified within the significant regions detected by the DCMS method.*

**TABLE 2:** Gene annotation for the significant genomic regions (autosomes) under putative selection identified by DCMS analyses in Sahiwal cattle.

Region (Mb)	*q*-value	Candidate gene	Trait
1:139.09–139.23 1:139.49–139.81	0.0058	KCNH, CPNE4, MRPL3, NUDT16, NEK11, and RF00026	Growth Milk protein content
3:65.95–66.60	0.018	ADGRL4, IFI44, IFI44L, RF00568, PTGFR, GIPC2, RF00026, DNAJB4, FUBP1, and NEXN	Milk yield Endothelial metabolism Antiviral property Heat stress response Adaptation Reproduction
6:17.56–18.29	1.76E-08	COL25A1, RF00156, ETNPPL, OST RPL34, LEF1, HADH, CYP2U1, SGMS2, and PAPSS1	Carcass traits Fatty acid, lipid metabolism Milk fat secretion Eye area pigmentation Immune regulation Mycobacterium resistance paratuberculosis
6:71.23–71.84	0.001	LNX1, CHIC2, GSX2, PDGFRA, KIT, and KDR LNX1, CHIC2, GSX2, PDGFRA, KIT, and KDR	Meat quality (meat tenderness, protein ubiquitination) Coat color
9:64.47–65.16	0.02	RF00099, RF000278, SYNCRIO, SNX14, and TBX18	Adaptation
9:10.21–10.22	0.01	RF00026, C9H6, PDE10A, TBXT, SFT2D1, MPC1, RPS6KA2, RNASET2, and FGFR1OP	Milk production Residual feed intake
11:94.29–94.36	0.03	OR5C1, OR1K1, PDCL, RC3H2, RF00579, ZBTB6, ZBTB26, RABGAP1, GPR21, STRBP, RF00026, CRB2, DENND1A, and RF00402	Reproduction
18:14.00–14.54	0.03	ZNF469, ZFPM1, ZC3H18, IL17C, CYBA, MVD, SNAI3, RNF166, CTU2, PIEZO1, CDT1, APRT, CALNS, TRAPPC2L, CBFA2T3, ACSF3, CDH15, SLC22A31, ANKRD11, SPG7, RPL13, RF00324, CPNE7, DPEP1, CHMP1A, CDK10, SPATA2L, VPS9D1, ZNF276, and FANCA	Milk yield and Mastitis resistance Reproduction Heat tolerance and Immunity
20: 21.99–2205 20: 22.14–22.42	0.0004 2.68E-05	RF00406, GPBP1, MIER3, SETD9, RF00003, MAP3K1, and RF00026	Milk protein and fat traits Survival
20:30.37–31.55	8.73E-07	MRPS30, RF00026, NNT, PAIP1, C20H5, TMEM267, CCL28, HMGCS1, NIM1K, ZNF131, RF00302, CCDC152, and GHR	Lactation persistency, teat and udder structure, Mammary function and mastitis Milk production
21:46.46–49.91	0.04	INSM2, RALGAPA1, RF00026, BRMS1L, MBIP, NKX2-1, NKX2-8, PAX9, SLC25A21, FOXA1, TTC6, SEC23A, GEMIN2, TRAPPC6B, PNN, and FBXO33	Reproduction Milk protein composition Lethal embryonic phenotype
23:39.17–39.67	1.79E-08	RF00001, RF00012, RNF144B, DEK, KDM1B, TPMT, NHLRC1, RF00026, KIF13A, NUP153, CAP2, RBM24, and STMND1	Bovine tuberculosis susceptibility Spermatogenesis Growth Body height Inflammation and immunity Stature

#### Putative Signatures of Selection on Other Chromosomes

##### Heat tolerance

The indigenous cattle (*B. indicus*) are best known for their heat tolerance among the tropically adapted species. The putative signals in our study were also identified on BTA 1, 3, 9, and 18. Among these *DNAJB4* is one of the functional candidate genes related with heat tolerance located on BTA 3, and this gene has a straightforward role in the cellular response during heat shock. This gene is associated with conserving the integrity of cytoskeleton, controls the protein folding, and removes the altered or misfolded proteins (Collier et al., 2008).

##### Coat color

Coat color in cattle is driven by complex molecular mechanisms and rendered it as a breed-specific characteristic in nature. In our study, we could identify three key genes *PDGFRA, KIT*, and *KDR* (Platelet-derived growth factor receptor alpha, KIT proto-oncogene, and Receptor tyrosine kinase insert domain receptor) located at 17-Mb regions on BTA 6. These three genes represent the *cluster of tyrosine kinase receptor* genes. In a study on the coat color patterns in the Nellore–Angus crossbred population, the red coat color is attributed to the three genes located in the region and is mostly associated with the *KIT* gene (Hulsman Hanna et al., 2014).

##### Milk and related traits

Sahiwal cattle along with other indigenous cattle are more revered in India due to their better milk attributes in terms of health perspective. Although studies on comparative milk profiles of Sahiwal and Holstein Friesian cattle suggested that milk composition of Sahiwal is slightly better than the Holstein–Friesian cattle in terms of fatty acid compositions (high unsaturated fatty acid 38.6%, low saturated fatty acid 61.4%, higher percent of monounsaturated and polyunsaturated fatty acids) (Sharma et al., 2018). The DCMS analyses in our study identified three genes, viz., Mitochondrial ribosomal protein L3 (*MRPL3*), Nudixhydrolase (*NUDT16*), and NIMA related kinase 11 (*NEK11*) located on BTA 1 at 139 Mb position. Interestingly, results of another study on weighted single-step genome-wide association analyses in Holstein and Holstein × Jersey crossbred dairy cattle coincided with this region (Raschia et al., 2020). This study associated *MRPL3*, *NUDT16*, and *NEK11* genes with milk composition traits. *ADGRL4* is an important functional candidate gene identified on BTA 3 related to the milk yield. Other studies on signatures of selection in the crossbred cattle reported a region that coincided with our study and is associated with milk production (Li et al., 2010; Singh et al., 2020). In our study, additionally one more candidate gene located on BTA 9 and related to milk production was observed (Mustafa et al., 2018). Multi-trait meta-analysis in Nordic cattle detected several loci with pleiotropic effects on milk production and mastitis resistance (Cai et al., 2020). We identified another candidate gene Zinc finger protein (*ZNF469*), associated with milk production and mastitis resistance in cattle in the intergenic region of BTA 18. *MAP3K1* (mitogen activated protein kinase kinase kinase1) is also known as *MEKK1* with single intronic indel codes Serine/Threonine kinase and was related to the *MAP3K* signaling pathway. It was also involved in breast cancer susceptibility in humans. Hence, it is assumed that this gene might play a role in the function of bovine mammary gland (Cuevas et al., 2006). Our analysis of selection signature could find out a candidate gene *MAP3K1* at 20 Mb on BTA 20, which is significantly associated with milk production traits in cattle. Two important candidate genes, Solute carrier family 25 member 1 (*SLC25A1*) and forkhead box A2 (*FOXA2*), are involved in the milk fat synthesis. SLC25A1 is known to be associated with the oleic and total monounsaturated fatty acid synthesis, and this gene is actively involved in two KEGG pathways such as metabolic and n-glycan biosynthesis pathways (Ibeagha-Awemu et al., 2016). Likewise, FOXA1 plays a role in the synthesis of cholesterol fat in the milk of cattle (Do et al., 2017). Copine7 (*CPNE7*), SPG7 matrix AAA peptidase subunit (*SPG7*), and FA complementation group A (*FANCA*) are a group of genes identified on BTA 18 and are associated with lipid metabolic, process cardiac system, and nervous system development. This region has a major pleiotropic effect in Chinese local cattle, which are diversified under adaptive selection (Xu et al., 2019).

##### Reproduction

Two candidate genes identified in this study (*DENND1A* and *NKX2-*1) were related to reproduction traits, while *DENND1A* was found to be associated with a number of embryos produced by the Holstein donor cows (Jaton et al., 2018). *NKX2-1* gene produces a transcription factor that controls the activity of thyroid-specific genes and is involved in morphogenesis. In a genome-wide association study, the *NKX2-1* gene is found to be associated with calving to first service and days open traits in Canadian Dairy Holstein cattle (Nayeri et al., 2016). These findings revealed the genetic control of the reproductive traits in the studied Sahiwal population.

##### Growth, survival, and adaptation

Copine4 (*CPNE4*), GIPC PDZ domain containing family member 2 (*GIPC2*), and Ral GTPase activating protein catalytic subunit alpha 1 (*RALGAPA1*) are the top candidate genes located in the peak value of the regions on BTA 1, 3, and 21, respectively, and are related to growth, survival, and adaptation, respectively. *CPNE4* is identified on BTA 1, and this region is associated with body size, muscle, and bone development in cattle (de Simoni Gouveia et al., 2014; Barbato et al., 2020). In a large multibreed genome-wide association study on milk production in dairy cattle, MIER3 gene was located close to the most significant SNP related to survival (Raven et al., 2014). Interestingly, this gene was also identified in our study. Sahiwal is a well-known indicine breed adapted to tropical environment and involved in the development of various synthetic breeds across the world (Australian Milking Zebu, Jamaica Hope, etc.). These synthetic cattle are also suitable for adaptation and resistance to tropical diseases (Ilatsia et al., 2012; Rehman et al., 2014). Temperature–humidity index (THI), a parameter combining temperature and humidity, is generally used along with other physiological variables, viz., respiration rate and rectal temperature, for quantification of heat stress in ruminants, including cattle.

In a genome-wide association study, *GIPC2* is located close to the most significant SNP, which is found to be associated with the adaptation in Columbian cattle (De León et al., 2019). In a similar manner, this gene was identified in our study, signifying its role in the adaptation of Sahiwal cattle for which they are better known.

### QTL Identification and Enrichment Analysis

**FIGURE 1:**
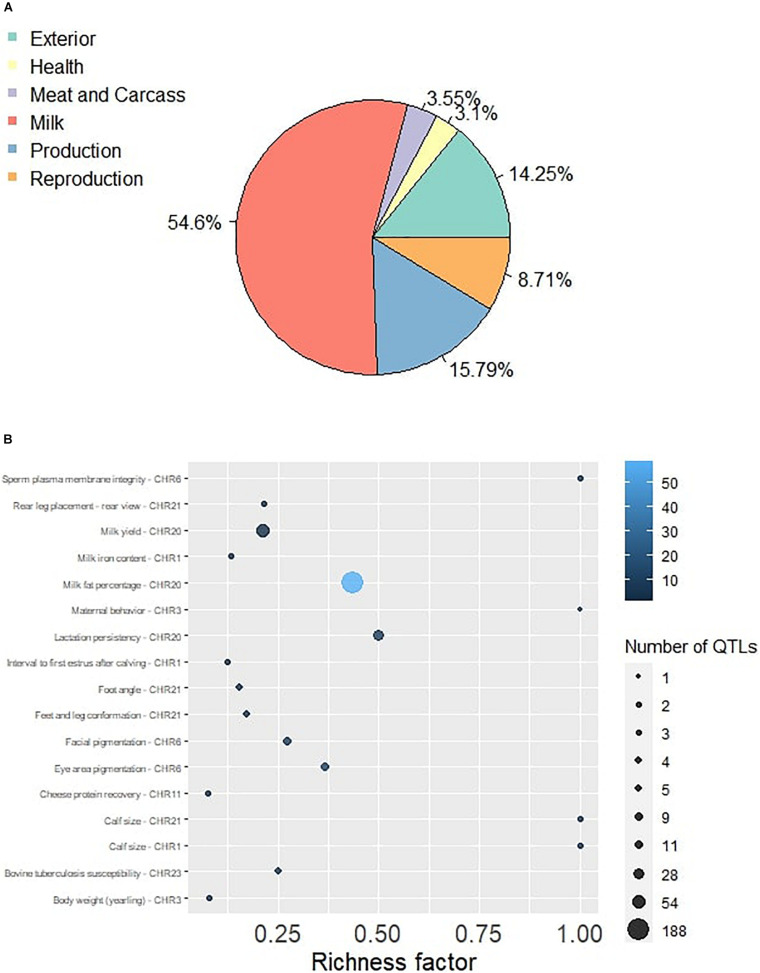
**(A)** The Pie plot showing the proportion of six QTL classes annotated in the significant genomic regions in Sahiwal cattle. **(B)** The QTL enrichment analysis determined the important traits enriched in the significant genomic regions. The number of observed QTLs for a class is based on the area of the circle. The color gradient denotes the *p-*value scale.

**FIGURE 2:**
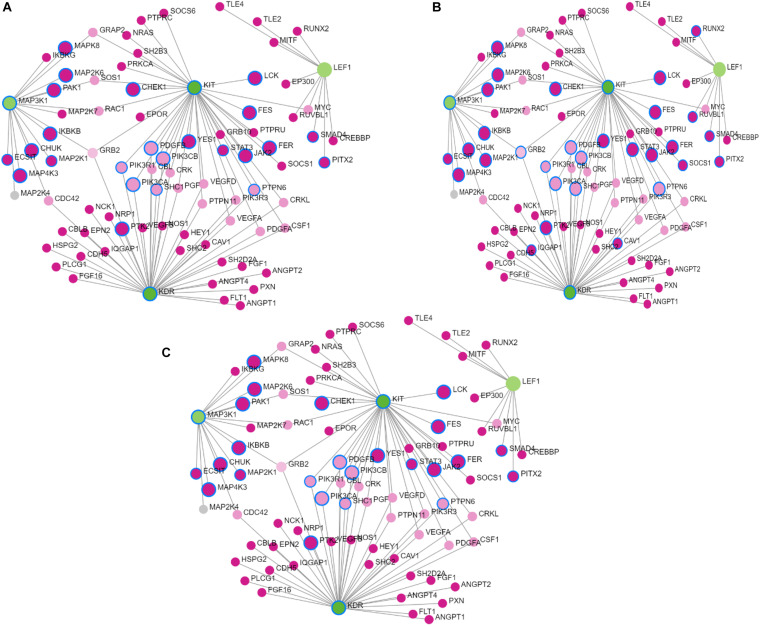
Results of Gene Network analysis for the selected prioritized candidate genes located in the significant genomic regions in Sahiwal cattle. **(A)** Biological process, **(B)** molecular function, and **(C)** cellular component. The green circles represent prioritized genes, while the pink circles denote the related genes.

The QTL identification revealed that significant genomic regions consist 54.6% of milk-type QTLs in Sahiwal cattle and other QTL types such as production, exterior, reproduction, health, and meat and carcass, which were annotated and accounted for 15.79, 14.25, 8.71, 3.1, and 3.55%, respectively (Figure 1). These QTLs were mapped to BTA 1, 3, 6, 9, 11, 18, 20, 21, and 23. The QTL enrichment analysis has shown 17 significant (FDR-corrected *p*-value ≤ 0.05) QTLs on chromosomes BTA 1, 3, 6, 11, 20, 21, and 23, which are associated with exterior, health, milk, production, and reproduction traits (Table 3). The top most significant QTLs were mapped on BTA 6, 20, and 23, which are associated with milk fat percentage, eye area pigmentation, lactation persistency, facial pigmentation, and bovine tuberculosis susceptibility (Figure 2).

**TABLE 3:** The enriched QTLs annotated in the putative genomic regions of the Sahiwal cattle.

Trait	Chromosome	QTLs (number)	Annotated QTLs (number)	*p-*value	FDR-corrected *p*-value
Exterior	6	11	175	1.73E-13	1.76E-11
	6	9	175	6.19E-10	3.15E-08
	21	4	70	0.000873	0.016187
	21	4	70	0.00141	0.023975
	21	3	70	0.002188	0.034331
	3	1	11	0.003696	0.044355
Health	23	5	11	3.88E-07	1.58E-05
Milk	20	188	467	7.06E-62	1.44E-59
	11	3	8	8.21E-05	0.002095
	20	54	467	0.000466	0.009506
	1	2	14	0.002738	0.03989
Production	20	28	467	7.86E-11	5.35E-09
	3	2	11	0.003558	0.044355
Reproduction	6	3	175	2.93E-06	9.97E-05
	1	2	14	2.71E-05	0.000791
	21	2	70	0.000372	0.008422
	1	2	14	0.003119	0.042418

### Gene Enrichment Analysis and Prioritization of Candidate Genes

The network constructed from the Network Analyst software consisted of 131 nodes and four candidate genes, KIT, KDR, LEF1 and MAP3K1, that are associated with skin pigmentation and milk fat percent. The gene ontology terms enriched in the analysis were GO: BP (124), GO: MF (46), and GO: CC (14), which are associated with lipid synthesis and skin pigmentation.

## Discussion

The present study is the first comprehensive report on genomic selection signatures wherein putative genomic intervals were explored using the DCMS method and major candidate genes for different traits of interest were identified in Sahiwal cattle. Selection signatures in Sahiwal were identified by the DCMS method, which combined the *p*-values from five different statistics into a new statistic, in contrast to the studies that considered the overlap in the genomic regions among the different methods. One earlier study utilized two complementary tests such as iHS and F_*ST*_ (Mustafa et al., 2018) to detect the loci under selection in Sahiwal cattle. However, the compound measure of DCMS gives precise and unbiased information about the genomic regions under selection by integrating the univariate statistic *p*-values (Grossman et al., 2010; Ma et al., 2015; Lotterhos et al., 2017). This method is helpful in accurate prioritization of candidate variants that will be useful to understand the mechanism of selection signatures that determine phenotypes in Sahiwal cattle. As a first step, we conducted IBD and PCA to check the presence of genetic clusters in the Sahiwal cattle. Our findings revealed that even if all the samples were homogeneous, they belonged to two subpopulations within the herd. Hence, we carried out the within group selection signature analysis.

Sahiwal cattle, which are distributed in the plains of Northern region of India, were under intense artificial selection for many generations. This breed is known for its potential for milk production and survives better even in harsh tropical climate. This breed could combat infectious diseases due to better immunity levels (Ilatsia et al., 2012). However, the information on the putative loci in the genome that controls these traits is not deciphered so far, which hindered our understanding regarding the mechanism of selection in these cattle. Genomic scans of Sahiwal cattle using the DCMS method captured a number of putative regions of selection associated with economic traits like growth, facial pigmentation, eye area pigmentation, milk production and composition, reproduction, body height, adaptation and survival. Our results suggested that the genome of Sahiwal cattle was under the pressure of recent ongoing selection.

Indian cattle breeds are categorically classified into three major groups based on their utilities, such as milch, dual purpose, and draught. Sahiwal is a well-known milch breed of India. Sahiwal has a lean conformation with brown coat color and better milk production attributes. In the present study, significant genomic regions and genes related to major economic traits under selection in Sahiwal were identified.

Herein, the four most significant genomic regions were identified in Sahiwal cattle: one each on BTA 6 (17567590:18290048, *q-*value = 1.76E-08) and BTA 23 (39175206:39671814, *q-*value = 1.79E-08), while two were on BTA 20 (30375415:30375415, *q-*value = 8.73E-07; 22144338:22425353, *q-*value = 2.68E-05). The most significant candidate genes identified in our study in the region BTA 6 (17567590:18290048, *q*-value = 1.76E-08) were collagen type XXV alpha 1 chain (*COL25A1*), Ethanolamine-phosphate phospho-lyase (*ETNPPL*), Oligo-saccharyltransferase complex non-catalytic subunit (*OSTC*), Ribosomal protein L34 (*RPL34*), Lymphoid enhancer binding factor 1 (*LEF1*), Hydroxy acyl-CoA dehydrogenase (*HADH*), Cytochrome P450, family2, subfamily U, polypeptide 1 (*CYP2U1*), Sphingomyelin synthase2 (*SGMS2*), and 3’-phosphoadenosine 5’-phosphosulfate synthase 1 (*PAPSS1*).

*COL25A1* gene is known as collagen gene located at 17 and 18 Mb regions. This gene is associated with carcass traits, and it is confirmed in a genome-wide study in Italian cattle breeds (Piedmontese, Marchigiana, Italian Holstein, Italian Brown, and Italian Pezzata Rossa breeds), as a similar peak is observed in Piedmontese beef cattle of Italy (Mancini et al., 2014). The *OSTC* gene is responsible for sperm membrane integrity in Holstein–Friesian bulls (Kamiñski et al., 2016). Cattle breeds were distinguished from each other due to specific pigmentation markings.

The pigmentation particularly around the eyes in cattle is termed as ambilateral circumocular pigmentation. This pattern makes those animals less susceptible to squamous cell carcinoma of the eye, and the *LEF1* gene is known for this eye pigmentation area in cattle (Pausch et al., 2012). *HADH* (Hydroxy acyl-CoA dehydrogenase), *CYP2U1* (cytochrome P450 family 2 subfamily U member 1), and *SGMS2* (sphingomyelin synthase 2) genes are associated with fatty acid and lipid metabolism, and thus help in fat globule secretion in milk (Li et al., 2010; Grilz-seger et al., 2019). The *PAPSS1* gene, which is associated with resistance to Johne’s disease, is also located in this region (Mallikarjunappa et al., 2018).

Our study could also locate another putative selection signature in Sahiwal, located on BTA 23 (39175206: 39671814, *q-*value = 1.79E-08) containing few important candidate genes related to bovine tuberculosis susceptibility, spermatogenesis, body height, and mineral content. The strongest candidates included Ring finger protein 144B (*RNF144B)*, Thiopurine S-methyltransferase (*TPMT*), and NHL repeat containing E3 ubiquitin protein ligase 1 (*NHLRC1*). The role of RNF144B toward bovine tuberculosis susceptibility was also reported in Holstein–Friesian cattle (Raphaka et al., 2017). This gene is associated with the expression of Nuclear-factor-kappa-B-inhibitor alpha (NF-*k*B) in human macrophages and also controls the function of various genes involved in a wide range of cellular processes like inflammation and immunity. In addition, it controls the apoptosis and cell proliferation. This protein coding gene is also conserved in other species, especially in humans (Zhou et al., 2016). It is important to note that the gene *TPMT* located on BTA 23 at the 39-Mb region is associated with porcine infections and affects the susceptibility to parasitic burden in pigs (Gaur et al., 2014). The tropical climate favors the prevalence of parasitic infections. These gene polymorphisms could affect the growth and production performance. Thus, this candidate gene could play a role in cattle production systems. Other important genes located on BTA 23 in Sahiwal cattle were NHL repeat containing E3 ubiquitin protein ligase 1 (*NHLRC1*) and family with sequence similarity 8 member A1 (*FAM8A1*). These functional candidate genes identified in this location were known to be associated with body conformation traits like body stature and mineral content, especially magnesium (Tizioto et al., 2015; Yan et al., 2020).

Another genomic region predicted to be under putative selection was located on BTA 20 at the 30-Mb interval, and a panel of candidate genes identified in this region were Mitochondrial ribosomal protein S30 (*MRPS30*), C–C motif chemokine ligand 28 (*CCL28*), 3-hydroxy-3-methylglutaryl-CoA synthase 1 (*HMGCS1*), and Growth hormone receptor (*GHR*). *MRPS30* is also known as programmed cell death 9 (*PDCD9*) with a role in breast cancer susceptibility (Fletcher et al., 2011). This gene has a role in cell apoptosis and found to be associated with lactation persistency in cattle (Appuhamy et al., 2009), whereas the *CCL28* gene is associated with the health of mammary gland by homing and inundation of IgA antibodies during the early lactation period (Hieshima et al., 2003). The *GHR* gene affects the milk yield and the lactation process by controlling the activity of growth hormone (Moisio et al., 1998; Rahmatalla et al., 2011). Similarly, the *HMGCS1* gene is associated with lactation persistency in cattle as this gene affects the synthesis of milk cholesterol and lipid (Rikitake et al., 2001; Murray et al., 2003).

### QTL Identification and Enrichment

The major fraction of QTL annotation in our study belonged to “Milk” type, which accounts for 54.6% of the total QTLs. The milk-type QTLs comprise milk protein percentage, milk yield, milk glycosylated kappa-casein percentage, milk protein yield, milk stearic acid content, milk protein content, milk margaric acid content, milk arachidic acid content, milk caprylic acid content, milk mid-infrared spectra, milk oleic acid content, milk lactose yield, milk phosphorylated alpha-S2-casein percentage, milk pentadecylic acid content, cheese protein recovery, milk *cis*-9-Eicosenoic acid, milk *trans-*10-Octadecenoic acid, and 305-days milk yield. The QTL enrichment analysis was performed to obtain the unbiased information about the significant QTLs present in the population rather than simply performing QTL annotation. The top five significant QTLs enriched are located on BTA 6, 20, and 23. The QTLs enriched include milk fat percentage, eye area pigmentation, lactation persistency, facial pigmentation, and bovine tuberculosis susceptibility. These results are consistent with our gene annotation findings, where the genes related to these characteristics were observed in the significant genomic regions. Sahiwal cattle are managed under selective breeding in the germplasm unit with an objective to improve the milk production and composition traits. The average milk yield in Sahiwal cattle is in the range of 1,500 and 3,000 kg. However, the production performance of a few top yielders is up to 4000 kg in a single lactation under organized herds. The milk fat in Sahiwal cattle ranges from 4.6 to 5.2% and SNF ranges from 8.9 to 9.3% (Joshi et al., 2001). The present QTL enrichment analysis also revealed that milk fat QTLs were annotated (1.44E-59) in the population. Interestingly, the top significant QTL identified on BTA 20 in our study is related to the milk fat percentage. The coat color and pigmentation patterns were breed specific in *B. indicus* cattle. The coat color in Sahiwal is one of the desirable characteristic features. Coat color might be under selection by breeders for a longer period. The findings of QTL enrichment are in the same direction. According to the literature, cattle breed with white color coat at the facial region are more susceptible to the squamous cell carcinoma (Guilbert and Wahid, 1948) compared to others, as they are more exposed to UV radiation.

Bovine tuberculosis is an important zoonotic disease in cattle, which causes huge economic losses globally. Srinivasan et al. (2018) examined the incidence of bovine tuberculosis in India through a meta-analysis study, which revealed that approximately 20% of cattle population were affected by this disease. Our study could find out major genes and QTLs associated with bovine tuberculosis susceptibility in individuals.

### Prioritized Functional Candidate Genes

The functional enrichment analysis of the top 20 prioritized genes revealed significant gene ontology terms that include biological processes, molecular function, and cellular component related to coat color, eye area pigmentation, and fatty acid and lipid metabolism. The *KIT, KDR, LEF1*, and *MAP3K1* were the top candidate genes prioritized, which are associated with coat color and milk fat percent in Sahiwal cattle. The findings in our study are helpful in comprehending the genetic control of a number of traits in Sahiwal cattle. This information is also useful in warranting the better genomic prediction for economic traits in Sahiwal cattle. The findings of our study also pointed out that the genes mapped onto BTA 6 and 20 had pleiotropic effects and had significant associations with other relevant economic traits.

## Conclusion

In summary, we have identified 14 significant genomic regions that are representative of putative signatures of selection in Sahiwal cattle. The methodology of DCMS was used in the computation of *p*-values from different univariate statistics into a composite signal, and the most significant regions were mapped onto BTA 6, 20, and 23. Our results are consistent with the domestication and selection history of these cattle. BTA 6 harbors the selection signatures, consisting of key candidate genes that are associated with coat color, milk fat percent, sperm membrane integrity, and carcass traits. These findings corroborate with the objectives of the genetic improvement program in the Sahiwal herd, where the purebred Sahiwal cattle with higher breeding values for milk composition and production traits are selected to produce the next-generation offspring. Similarly, two putative genomic regions at the intervals of 22 and 30 Mb on BTA 20 are found to contain genes associated with the variation in milk fat, milk yield, and lactation persistency in these cattle. Another important region on BTA 23 at the 17-Mb region harbors genes related to susceptibility to bovine tuberculosis and tolerance to parasitic infestations. The highly significant genomic regions helped in shaping the demography and trait architecture in this breed and established it as one of the most suitable milch cattle breeds in the tropics. Prioritization analysis could identify key candidate genes that control pigmentation and milk fat percent. Our study reveals several loci that are associated with quantitative traits, disease resistance, and adaptation to tropical climate in the Sahiwal genome that might have been affected by years of selection. The present investigation highlights the importance of *B. indicus* cattle, which perform better in terms of both production and adaptation to tropical climate due to their unique genetic mechanism that is deciphered. The future looks more promising since the implementation of this genomic information in the ongoing breed improvement program might well enhance the genetic progress in Sahiwal cattle.

## Data Availability Statement

The raw data supporting the conclusions of this article will be made available by the authors, without undue reservation.

## Ethics Statement

The animal study was reviewed and approved by Institute Animal Ethics committee, NDRI (NDRI approval 43-IAEC-18-8).

## Author Contributions

AM and SM had conceived the idea. SI, SN, and SM performed the entire bioinformatics analysis. SI, AM, and SN wrote the manuscript. SM done editing and proofreading. AM supervised the entire project. All authors read the manuscript and approved the final manuscript.

## Conflict of Interest

The authors declare that the research was conducted in the absence of any commercial or financial relationships that could be construed as a potential conflict of interest.

## Footnotes

1 https://github.com/ngarud/SelectionHapStats
